# Supplementary material for: Pre- and postsynaptically expressed spike-timing-dependent plasticity contribute differentially to neuronal learning
Source: PLoS Comput Biol. 2022 Jun 14;18(6):e1009409. doi: 10.1371/journal.pcbi.1009409 (PMC9236267; doi:10.1371/journal.pcbi.1009409)
Supplement: S1 Appendix — (PDF) [file pcbi.1009409.s001.pdf]

S1 Appendix — Rate Model

Using a simple firing rate model with linear response, we were able to illustrate how synaptic plasticity could separate correlated and uncorrelated inputs without competition between the two populations. Considering a neuron receiving independent Poisson inputs with fixed firing rate (pooled into a single average input $I(t)$), we found that the system tends to a specific non-zero average value for P, denoted $P*$ below. We converted the biophysically tuned model (eqs. 10 and 11) to a firing rate representation with time-averaged values:

$$< dq >= c^{+}\tau_{x_+}\tau_{y_-}I\nu^2 \quad , \tag{1}$$

$$< dP >= \nu I\tau_{y_+}(d_+\tau_{x_+}I - d_-\tau_{y_-}\nu) \quad . \tag{2}$$

Postsynaptic output ν was then considered as a simple firing rate model with linear relation to average input I, weighted by average synaptic efficacy:

$$\nu = \alpha + \beta qrpI \quad . \tag{3}$$

To determine α and β values that corresponded to the simulated neurons (for fixed values of q and P), we fitted to data from simulations without plasticity. Since I was fixed, we could also consider stationary values for $r(t)$ and $p(t)$, $\bar{r}$ and $\bar{p}$, from eqs. 15 and 16:

$$\bar{r} = \frac{1 + PI\tau_F}{1 + PI\tau_F + PI\tau_D(1 + I\tau_F)} \quad , \tag{4}$$

$$\bar{p} = \frac{P(1 + I\tau_F)}{1 + PI\tau_F} \quad . \tag{5}$$

We thus have $< dq > (P, q, I)$ and $< dP > (P, q, I)$ in the LTP equations 17 and 18:

$$\nu \approx \alpha + \frac{\beta qIP(1 + I\tau_F)}{1 + PI\tau_F + PI\tau_D(1 + I\tau_F)} \quad . \tag{6}$$

We plotted $dP \times dq$ as a vector field (Fig S1A), which shows how P tended to the specific value $P*$, which corresponds to the average value of P for uncorrelated inputs. Note there is no specific $q*$ value as q only increases. Integrate-and-fire simulation averages also converged to this point (black line, Fig S1A). This is in contrast to correlated inputs, which potentiated more (Fig 6). The value $P*$ was relatively stable with frequency, but saturated at a limited frequency value(Fig S1B), effectively limiting the range of possible separation between the correlated and the uncorrelated populations. It is remarkable that for frequencies below this limit the total weight value $W = Pq$ doesn't grow unbounded with positive feedback, even if the postsynaptic side q saturates.

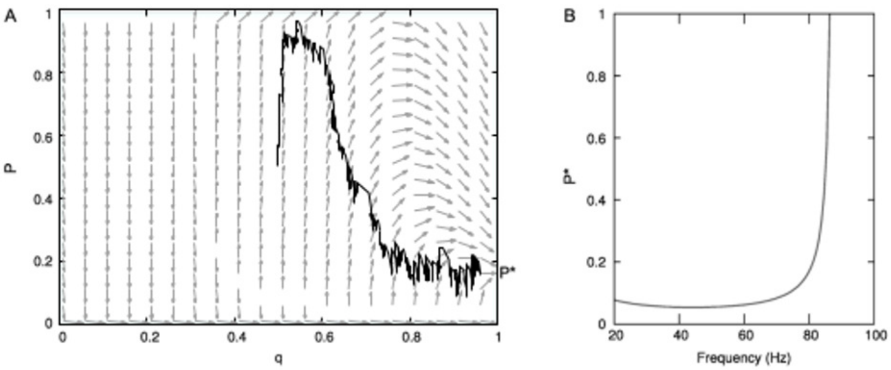

Fig S1. Plasticity separated correlated and uncorrelated inputs up to a limiting frequency. (A) Vector field (p x q) representing the rate model for uncorrelated synaptic inputs only. The black line shows corresponding integrate-and-fire simulation averages for uncorrelated inputs (compare Fig 6A). Note how both models converge to the same fixed point, indicated with the label $P*$. **(B)** The point of convergence $P*$ was relatively stable with respect to presynaptic frequency up to a limiting frequency of around 85 Hz, where it saturated. Since correlated inputs tended to saturate, this shows an effective upper frequency limit to the clustering of correlated and uncorrelated inputs.
