# Supplementary material for: Pre- and postsynaptically expressed spike-timing-dependent plasticity contribute differentially to neuronal learning
Source: PLoS Comput Biol. 2022 Jun 14;18(6):e1009409. doi: 10.1371/journal.pcbi.1009409 (PMC9236267; doi:10.1371/journal.pcbi.1009409)
Supplement: Attachment — Submitted filename: Mizusaki_rebuttal.pdf [file pcbi.1009409.s002.pdf]

Rebuttal for Mizusaki *et al*, PCOMPBIOL-D-21-01572

We were delighted to learn that the reviewers were quite positive about our revised manuscript. Again, we think our manuscript was strengthened considerably by the reviewers' suggestions.

Here is a summary of a few key improvements:

1. We extensively rewrote the results section, highlighting which presynaptic characteristic was explicitly modelled in each simulation.
2. We revised the methods section, adopting suggestions to simplify some nomenclature and indicating the meaning of all variables referenced.
3. We clarified the results section breaking down our main conclusions into separate points.
4. We integrated suggestions to make figures more interpretable.

Reviewer #1

The manuscript addresses a relevant and interesting questions, aiming to distinguish the effects of changes in synaptic strength via increase/decrease in presynaptic vesicle release probability versus via an increase/decrease in postsynaptic receptor density. The former process impacts the short-term dynamics of vesicle release, such that long-term changes in synaptic strength are likely to have concomitant changes in the ratio of facilitation/depression. Since these changes that are overlooked in most/all models of plasticity in networks, the question is important.

We are delighted that the reviewer sees the relevance and interest in the question that our manuscript addresses and that they think this question is important!

Overall, the strategy used is clear and good, as the authors address a couple of roles of plasticity in a simple setting and compare models with and without the different contributions to changes in synaptic strength. I do find some of the specifics to be unclear though, in particular, I found insufficient description of the precise model when the authors attempt to isolate the facilitation/depression dynamics from the presynaptic strength-change – that is, how Fig 4 is achieved in comparison to Fig 3.

We are happy that the reviewer thinks our strategy is clear and good! But we can see how certain specifics remained unclear. On the presynaptic side vesicle, release happened with probability p which varies at ms timescales according to short-term plasticity. However, simplified phenomenological models of long-term synaptic plasticity commonly omit stochastic release altogether. Starting from this, we chose to separately study the first step of simply including stochastic release, to then add the more biologically reasoned dynamics of p. In the revised manuscript, we have improved the clarity of this point (see Results section, lines 116-124).

Fig 2 Caption and throughout: "The learning task" is a misnomer as there is no animal performing a task who can learn anything. Rather the paper is assessing how patterns of inputs give rise to different outputs in a circuit. There is no goal or "right answer". Moreover, connections do not learn they change even if those changes almost certainly underlying an animal's learning. Perhaps the paper could be reframed to particular tasks that animals must learn and the simple circuits be proposed to underly such learning. I don't think that is necessary and just an alteration in the language used would be fine.

We agree that calling this a “learning task” could be confusing. Reviewer 2 furthermore had a similar concern. We decided to call it “learning scenario” instead. We also speak of “outcome” instead of “task.”

l. 156 “We next explored the effects of altering short-term dynamics”. The statement is confusing as the previous section already explored these effects via changes in “p”. Do you mean “altering short-term dynamics *without* any concurrent change in synaptic strength? Or do you mean while the change in synaptic strength is matched to the postsynaptic plasticity? It is really unclear to me, even when looking over the methods. Moreover, however this is done, how doe “postsynaptic alone” in this section (and Fig 4) differ from that in the prior section (and Fig 3) since “postsynaptic alone” could not impact short-term dynamics in either section. The “combined pre and post” becomes even more complicated to think about as distinct from the prior section or from “post alone” if short-term dynamics are present/missing. All rather confusing, so please clarify in the main text as well as the methods!

Thanks for highlighting this confusion. When we first model changes in vesicle release probability, we deliberately omit changes in short-term plasticity.

The rationale comes as we started looking from an abstract concept of synapse: an input-output relation between pre- and postsynaptic neurons’ activities. Most phenomenological models consider this function deterministic and ignore shorter time dynamics in relation to LTP. We know that a single synapse responding to a single action potential is an unreliable information channel. We decided to first test the impact of simply the unreliability itself, without including dependencies on recent activity. This would be equivalent to STP being in a stationary state. In the next step, some biological reasoning was included as we considered a ‘baseline’ release probability (P) and have the instant probability (p) vary according to the Tsodyks-Markram model. We tried to clarify this in the original manuscript (lines 107-108) when we said, “To distinguish between the impacts of stochastic transmission and short-term plasticity, we first model the synapse without STP.” This was done to distinguish the effects of changes in synaptic noise levels from those due to changes in synaptic short-term dynamics. Therefore, the postsynaptic-only forms of plasticity are in effect different in the two scenarios; the former is limited to effects of stochastic release, whereas the latter includes short-term modulation according to recent activity.

Early in the project, we realized that solely modelling presynaptically expressed plasticity as changes in short-term dynamics without accounting for stochasticity would omit key aspects of synaptic release, i.e., the changing noise levels.

We clarified this on lines 102-111, 116-118, and 165-167 of the revised manuscript.

l. 198-214. There seems to be some confusion in the text here. If “LTD is solely postsynaptically expressed” then why in l.211 does “postsynaptic plasticity lack the ability to depress”?? And those problems resolved by inclusion of “presynaptic LTD” in the biological model if LTD is not presynaptic? I think some switching of “pre” and “post” in the text is needed and ne sure it matches Fig 5.

Sorry, there was an unfortunate typo on line 199 of the original manuscript, where “LTD is solely postsynaptically expressed” should have been “LTD is solely presynaptically expressed.” Thanks for catching this! We fixed the typo, and we furthermore edited the subsequent paragraph for clarity (lines 208-222).

Fig 6. It is confusing that for the red and the blue traces in A there is essentially no difference between the “unc” and the “corr” results so there is no clear reason as to how inputs are segregated?

And how is this compatible with the classic papers of Song & Abbott showing separation of strengths for “corr” and “uncorr” inputs with postsynaptic plasticity alone? (Blue lines in your figure converge to identical values).

Correct, the inputs only segregate when both pre- and postsynaptic plasticity are simultaneously engaged. The biologically tuned model lacks capacity for postsynaptically expressed LTD (see the above unfortunate typo), so this outcome is in fact correct when presynaptic expression is abolished. This model — which closely captures pre and postsynaptic expression of STDP — can then not properly recapitulate Song & Abbott’s findings. We think this is a valuable observation.

And if weights are kept constant in the red/blue traces then why are they not kept constant in the black trace (far right)? All rather confusing.

This is in fact correct. The colors indicate the mode of plasticity, with red being the simulation where q is kept constant, blue the simulation where p is kept constant, and black the full model, where both p and q change. Each graph illustrates the values of a synaptic variable: on the left, it is p (thus the blue trace remains constant), in the middle, q (red trace kept constant), and on the right, it is the product of p and q (no trace is kept constant). However, it is visible that value convergence is faster and “cleaner” when only one variable is changing. The interplay between p and q dynamics results in a state that is unstable, thus it constantly fluctuates while receiving input. We clarified this in the revised Fig. 6 caption.

l. 317 “considerable impact on the learning outcome”: Maybe I missed this, but I saw no overall difference, just a change in the speed at which changes arose in the correlation paradigm – though a change in latency was a difference, but I think “small impact” with no qualitative change and it is hard to see what “learning outcome” for an animal is particularly impacted here if you wish to match these results to learning.

The reviewer did not miss it; it was part of the confusion just above. Among the outcomes was that postsynaptic change is much more efficient in encoding time and output gain. This makes it interesting to consider when studying a specific setup where it is found that LTP is mostly presynaptic, for example. In this case, it would be unlikely that specific time intervals or large changes in output frequency were functionally relevant for that connection. This is an example of the “considerable impact on the learning outcome” that we speak of in the Discussion. We clarified the Discussion on lines 328-338 to better highlight this surprising outcome.

l.343-4 Eq.2: Why is the conductance only impacted by “q” rather than release probability “p”? Especially since in the rate model the connection strength “W” is proportional to both “p” and “q”.

We have reworded the paragraph (lines 358-362) and Eq. 2 to make sure the effect of “p” is clarified. Briefly, inputs were received with probability p_j, so conductance was not impacted solely by q_j.

l. 367-8 Eq.5: Why is the function “F” a constant rather than exponentially decaying? As written the rate of change of W depends on all prior (and future) spikes throughout all of time as they each contribute the same constant amount. This seems unphysical.

This was a mistake, thanks for noticing. It has been corrected in the manuscript.

l. 376-7 “When the rate convergence rates were compared” It is unclear how you are comparing them when you are forcing them to be identical as you say you “ensure” they were “the same for all simulations”?

What is ensured is that under the exact same pre- and postsynaptic activity, the ΔW outcome is the same. However, a change of the same magnitude in p or q does not affect postsynaptic activity equally (see Fig. 1B) and thus subsequent ΔWs develop differentially due to the different history of activity.

Minor:

Fig 3 caption: two “and”s near the end.

Done.

l.136 just “2B” I think

Correct, this was changed.

l. 185 “I”

Done.

l. 192-3 “relevance … was unclear”

Done.

l. 318 “these effects”

Done.

l. 377 I assume that the superscripts “f” and “i” denote “final” and “initial” here and elsewhere, but I don’t see where these are defined.

This was changed to “t” and “t+1”.

Fig 7. I suggest you make the arrows smaller so that they are separated – it is hard to tell which way they point when they run into each other, and the change in direction of the flow fields is important.

Thanks, they are now slightly smaller.

Reviewer #2

Here the authors present simulation results illustrating how the effects of spike-timing-dependent plasticity can vary depending on whether it is expressed pre- or postsynaptically. They focus on two scenarios: 1) a latency paradigm, where early inputs potentiate and late inputs depress to reduce the latency of responses of a postsynaptic neuron, and 2) a correlation paradigm, where a set of correlated inputs potentiates and sets of uncorrelated inputs depress causing the postsynaptic neuron to covary with the correlated inputs. In simulations of stochastic release, short-term plasticity, and a biologically tuned model they find that the locus of expression can alter the speed of potentiation/depression and several other outcomes for each paradigm.

This is a well-structured paper that makes a strong argument for the importance of thinking about locus of expression with plasticity. It develops interesting new ideas, and it will certainly be interesting to many computational and experimental neuroscientists.

I have no major concerns. The logic and arguments are clear. However, I did find the results section somewhat difficult to understand. Some details seemed missing or could otherwise be more clearly described here (see multiple points below).

We were delighted that the reviewer thinks our paper is well structured and that it makes strong arguments! We were also happy to see that the reviewer thinks our paper develops interesting new ideas that will be of interest to a range of neuroscientists of different background. We are very happy to see that there are no major points but only minor comments regarding clarity.

Minor Issues:

Line 95: “First, in the reliability of transmission due to stochastic vesicle release.” Incomplete sentence?

Thank you, this was fixed.

Line 96: “keeping the standard deviation roughly the same” This seems unnecessarily vague, since you can write down the change in CV analytically. Why not just say that the standard deviation decreases (when initial $p>0.5$) or increases by a smaller amount than the mean

Thank you. Reviewer 3 made a similar comment. We modified the text (lines 95-111).

Line 110: “we model presynaptic expression as changes in short-term plasticity and compare that postsynaptic expression” Missing “to” unclear what “that” refers to.

Correct, thanks! Reviewer 3 also had related comments on this section. This has been clarified (lines 95-111).

Line 119: “Here, changes in pj were explored in terms of their impact on stochastic release” … the previous sentences make it seem like p and q will both be varied. I would also suggest spelling out what j is

Right, thank you. The two introductory sentences were accidentally kept from a previous version of the manuscript, so were removed.

Line 120: It may be helpful to have add some context here. E.g. “With the latency paradigm, STDP leads early inputs to potentiate and late inputs to depress.”

Thank you, we added this sentence.

Line 129: What is “variance of the latency shift” referring to? Variance across simulations at a specific time point? The variance is difficult to see in the figure alone.

We mean variance across simulations. The text was modified.

Fig 3 caption: “Time evolution of average synaptic weight among early and late presynaptic inputs (i.e., input cells that spiked in the first or the second half of the stimulus) show how post-only expression (blue) is relatively slower.” … This seems incomplete – isn’t postsynaptic expression slower for potentiation, faster for depression?

Good point, this is correct. It is, however, the early group that drives the development of the outcome in this learning paradigm, so it is still a fair statement. We clarified the text.

Fig 3: Suggest maybe using dashed lines to make it clear that pre follows pre+post in panel F

This is a good suggestion, thanks. We have now done this Fig. 3F.

Fig 3H: It would be helpful to add “corr” and “unc” labels here, as well as an indicator that c>0.9 here. I would also suggest adding the value of c for 3G for context.

Good idea, labels were added! Because 3G was implemented as to replicate the Song & Abbott paper, the specific value of c is not well defined. For the rest of simulations, we used a different method to generate correlated inputs (see line 384, ref. 68, Brette 2009), with which we had direct control over c values.

Fig 3I: a colorbar would be helpful here. Is this the difference in slopes? Weights? The caption seems to suggest it’s related to “which side potentiated faster”, but the text seems to focus on who “won”

Thanks, this is a good suggestion. A color bar was added, and the Fig. 3 caption was clarified too.

Fig 3I caption: typo “and and”

Done!

Line 140: “was faster for strongly correlated input firing at certain input frequencies”. Should clarify that it’s faster for both correlated (faster to potentiate) and uncorrelated (faster to depress) inputs? Additionally, it’s not clear what the “input frequency” is here or that it matters.

Correct. We edited the text accordingly and further clarified.

Line 150: “we systematically explored the correlation-frequency space”. It’s somewhat unclear what this means. It may be useful to spell out that (if I understand correctly?) these are distinct simulations where all inputs have a specified correlation and firing rate.

Yes, that is correct — are distinct simulations where all inputs have a specified correlation and firing rate. We clarified the text accordingly (lines 152-154).

Line 152: “a scenario that corresponds to fewer inputs spiking synchronously” I’m not sure what exactly this is comparing.

This comparison was confusing and added little of value, so we removed it.

Line 167: “more subtle” I wasn’t completely sure what this meant. Just that the change in latency was smaller?

Yes, that is correct. We edited the text as suggested.

Fig 4 caption: “between p and q” and “between p and both”. Suggest spelling out pre or postsynaptic expression here to avoid confusion.

Good point, p_{release} could be confused with $p_{\text{t-test}}$. We modified as suggested.

Line 182: “STP modulation” I guess this if the first use of the acronym. Could spell it out for clarity here.

It turned out we only used the “STP” abbreviation twice in the manuscript, so we opted to remove it entirely and instead spell it out as “short-term plasticity”.

Line 185: typo "I"

Fixed, thank you.

Line 187: "It thus appears that computational advantages could be tailored to a functional task at hand by recruiting pre- or postsynaptic plasticity differentially." I'm somewhat confused by this conclusion. Unlike the results from Fig 3G, it seems like there's minimal difference between the speeds when the dynamics are included. I'm unsure if/how 4G and 3H can be compared directly, but at low firing rates it also appears that the site of expression doesn't matter.

Thanks, this is a very good point. The text was not structured correctly to bring home this message well enough. I think the better comparison is between Fig. 4B and 4F — relatively speaking, the correlation paradigm is not much affected by the locus of expression, but the outcome of the latency shift is. In conclusion, the impact of the locus of expression depends on the computational task at hand. The text was reworded accordingly.

Line 206: "As with the above simplistic modelling scenarios" I guess this is just the case with dynamics.

By "simplistic modelling scenarios," we meant the toy models that were not biologically tuned. We edited the text for clarity.

Line 207: "spike timing" Might specify that it's "latency", since other aspects of timing aren't directly analyzed?

Agreed, this was changed.

"when both pre- and postsynaptic plasticity were active, the presence of postsynaptic potentiation further reduced the latency compared to presynaptic plasticity alone." This seems like an odd framing for modelers who traditionally only use postsynaptic plasticity. It may be useful to reframe by focusing on the fact that pre+post expression is much slower than post alone.

Fair enough. We rephrased the text as suggested.

Line 225: It's not clear to my why one would classify based on p rather than w.

Sorry, this was a typo; it was done on W, as was correctly stated in the Methods section.

Line 283: "It is also interesting to think about the role of presynaptic plasticity if it is not very useful in the context of usual 'coding' frameworks." To the extent that STP acts as a filter or mechanism for gain control wouldn't presynaptic expression of long-term plasticity just reflect changes in the filter/gain control? This seems like a direct impact on coding.

This is a good point. We removed this sentence.

Line 352: typo "Peduction"

Done.

Line 364: typo "implemente"

Done.

I couldn't find how many inputs were used here or how many simulations are being averaged over in the results. I would also suggest explicitly describing the error bands/bars in the captions of figures where they appear (S.D., C.I. or S.E.M?).

We edited the captions to include this information.

Reviewer #3

In their paper Mizusaki et al address an important and timely question of how the site of expression of synaptic plasticity affects the outcome of learning. While we do know since work of Tsodyks & Markram that pre- and postsynaptic changes modify cell's responses to irregular inputs in completely different ways, detail of these differences and their computational consequences remain poorly explored. Systematic analysis of such differences would make an important contribution toward understanding specifics of learning in diverse neuronal networks.

We thank the reviewer for pointing out the importance and timeliness of our study, and for highlighting how the computational consequences of presynaptically expressed long-term plasticity remains poorly explored!

However, while the topic of the study is interesting, and the paper could potentially make an important contribution to the field, there are several concerns that should be addressed.

In my opinion, major problems that needed to be addressed are:

1. Description of results (actually methods too) is neither precise nor complete; there are numerous unsubstantiated or unclear statements (specified below). Also, in the present version it is not clear whether and how model settings were changed in different experiments (see below); this should be clearly described.

We are sorry for coming across as unclear or imprecise. As detailed below, we addressed all the points raised by Reviewer 3.

2. Modeling presynaptic changes that do not lead to changes of synaptic dynamics looks odd. The rationale for using such a model, which contradicts almost everything we know about synaptic transmission, should be clearly articulated.

As explained above (Reviewer 1), this was a conceptual but important point. From a minimalistic point of view, we simulated the simplest functional separation between pre- and postsynaptic weights. That meant presynaptic weights controlling the reliability of transmission and postsynaptic weights controlling the amplitude of the signal. It can be thought of as if the short-term synaptic dynamics were in a stationary state. So that is where we situated the first model, as an exploration of the concept of changes in noise x amplitude. Note also that this artificial scenario of changes in p without changes in short-term plasticity is only studied in the first toy model, which we explicitly state that is not meant to be biologically realistic. Of course, when bridging the relationship with biological compatibility, we subsequently move on to introduce the impact of changes in short-term dynamics. We thus do not ignore short-term plasticity.

Finally, the classic STDP studies by Song & Abbott (2000, 2001) — which form a strong foundation as well as a source of inspiration for our present study — had neither short-term plasticity nor stochastic release. So, we believe the implementation of stochasticity independently to short-term plasticity in our study is well justified as a methodologically sound extension to the prior literature.

In the revised manuscript, we provide a better rationale in several places (e.g., lines 86-124 and elsewhere). We also highlighted that ours can be considered an extension to the classic STDP studies by Song & Abbott (Song et al 2000; Song & Abbott, 2001) in the Discussion (lines 329-331: "To do so, we relied on two classic STDP studies…").

3. Results of simulations with Latency paradigm and Correlation paradigm should be presented to allow comparison. e.g. there is a lot of description of spiking response in latency paradigm, but not in correlation paradigm experiments.

These two paradigms were deliberately chosen because they are quite distinct from each other and should not be directly compared. In fact, the original studies on which we based these two scenarios were published in two different papers (Song et al 2000; Song & Abbott, 2001). In the Latency Paradigm, activity comes in short bursts, similar to how gratings might drive activity in visual cortex (Song et al, 2000). In the Correlation Paradigm, however, spiking is relatively continuous and random, but is correlated among half of the inputs, thus emulating a scenario where a subset of inputs carries information (Song & Abbott, 2001). It is therefore not possible to provide a latency measure for Correlation Paradigm, and it is not meaningful to report on correlations in the Latency Paradigm.

Specific comments:

l.55 'depletion' – double

Thanks! This was fixed.

l.96-98 "Assuming release is binomially distributed, increasing the probability of release, p, increases the mean of synaptic responses while keeping the standard deviation roughly the same, ..." – this is plainly wrong. With N=5 (Methods, l.408) and q=1; with p= 0.1, 0.2, 0.3 the mean is 0.083, 0.167, 0.25 and SD is 0.133, 0.197, 0.248. How 0.133, 0.197, 0.248 are "roughly the same"?

Sorry, this was a confusing statement. We meant to say that relative to the mean, the standard deviation remains relatively stable as the probability of release is increased. It is thus the change in the SDev that should be compared to the change in the mean, not the SDev that should be compared to itself. For example, if we fix n to n=5 release sites, the mean equals 5p, whereas the SDev equals sqrt(5*p*(1-p)), so for biologically relevant values of p, the CV is decreased if p is increased, indicating that synaptic noise was reduced. This is the foundation of the CV analysis method for finding the locus of expression in long-term plasticity experiments, e.g., see classic paper by Faber & Korn Biophys J 1991 60:1288, which is why we self-cited Brock et al (2020), where we describe this approach in detail. In the revised manuscript, we improved the discussion of the impact of changes in p on synaptic noise levels on lines 102-111.

l. 108-112 "We start with presynaptic expression modelled as direct changes in the probability of vesicle release and compare that to postsynaptic expression. Subsequent to that, we model presynaptic expression as changes in short-term plasticity and compare that postsynaptic expression."

Not clear what's the difference. If 'direct changes of p' means no short-term plasticity, it's a mathematical abstraction without any biological sense. At least I am not aware of a cortical synapse that does not express STP that depends on p.

Please either provide a clear rationale for using such a construct, or remove.

Thank you, this is an important point — please see our reply to point 2 above. This is indeed a mathematical abstraction without any biological sense. It is, however, not unimportant, because our modelling also has key implications for artificial implementations of neural-inspired networks. Typically, these rely on deterministic synapses, but there has recently been speculation that processing capacities could be introduced by using stochastic connections (e.g., ref. 61, Aitchison & Latham 2015).

We were not clear enough about the difference between P and p: the former is the fixed release probability and relates to the presynaptic contribution to the overall synaptic weight, W, whereas the latter is the instantaneous probability of release, which varies dynamically during high-frequency presynaptic activity. In other words, in the purely stochastic case, we set $p = P$, whereas with STP, we set $p = p(P,t)$. This is now clarified on lines 129 and 169-170.

Still, while all synapses may have a degree of short-term plasticity, not all have short-term depression. For example, while connections from layer-5 pyramidal cells onto neighboring pyramidal cells show short-term depression, those onto neighboring Martinotti cells show marked short-term facilitation across a wide range of frequencies (Blackman et al., 2013). We thus believe that it makes good sense to tease apart the contribution of changes in synaptic noise from the contribution of altered synaptic short-term dynamics.

As outlined in reply to point 2 above, we clarified the manuscript in several places.

Fig 2 legend.

"(A) ... The learning task is thus to reduce the latency and to shorten the duration of the postsynaptic spiking response"

" (B) ... The learning task is thus to select for inputs that are correlated at the expense of those that are not"

'Learning task' sounds weird here. Whose task?

What is actually described, is how inputs with two fundamentally different types of temporal structure are affected by STDP.

We agree that calling this a "learning task" could be confusing. Reviewer 1 furthermore had a similar concern. We decided to call it "learning scenario" instead. We also speak of "outcome" instead of "task."

"After learning, the postsynaptic spiking (blue raster at top) was more correlated with the correlated inputs (pink histograms) than with the uncorrelated inputs (red histograms), indicating that the former drove postsynaptic activity." Same was true before learning... It's kind of commonplace that more correlated inputs have stronger impact on postsynaptic firing, with or without plasticity.

Sorry, this sentence was not phrased correctly. We should have said that the output is more temporally correlated with the correlated inputs after learning than before. We rephrased the text.

Also, from this figure and its description it's not clear how two paradigms will be compared; How to compare a decrease of spike number and latency on the one hand, with a change of the correlation between inputs and spiking on the other?

The two scenarios are quite different and should not be directly compared. We changed the figure caption text accordingly.

l. 126. " decrease of postsynaptic activity duration and increase of postsynaptic firing frequency" – do not fit together.

Sorry, there must be some sort of misunderstanding here. These two claims do in fact fit well together, because that is what happens in the model: the response activity duration is decreased (Fig. 3D) and the intra-burst firing frequency is increased (Fig. 3E). Song et al (2000) reported similar findings (their Fig. 4B, D). No edits were made, but we are happy to modify the text to make it clearer, we just do not understand what we are supposed to change.

Fig. 3

3B: X-scale labels are missing. Why is it different from A, D-F?

The label is not missing, it says "Trial". There are no units because trial number does not have units. The x-axis labels in panels D-F are in fact not different compared to that of panel B; this must be a misunderstanding. Panel A illustrates individual trial sweeps before and after, so must have different x-axis label and units. We edited the panel A caption to try to clarify this distinction.

3G: what was correlation (strongly, weakly correlated inputs)?

In Fig. 3G, the correlated inputs reproduced the Song & Abbott paradigm, for which the value of c is not formally defined. However, considering the lack of synchronized inputs, one could classify the correlation as low. For the rest of simulations, we used a different method to generate correlated inputs (see l.367), with which we had direct control over c values. The strongly correlated inputs ($c>0.9$) were already described in Fig. 3H.

3B,D-F X-scale is in trials; in G,H – in s; is it possible to make them uniform? To somehow compare the rate of synaptic changes?

Sorry, no this is not possible, nor is it meaningful. These are different learning paradigms that were not carried out in a comparable manner. For example, the trials of the latency paradigm could in principle have any inter-trial spacing — just like in Song et al (2000), spaced versus massed learning is not something that the model cares about. See our reply to Point 3 above for additional information.

3I – not sure I understand what and how was going on here. All inputs were saturated but some faster, some slower? Please show examples of synaptic changes in two groups, for a 'red' and a 'blue' case.

Sorry, the caption for Fig. 3I was confusing. The color code indicates which half ultimately won out. But they won out because they potentiated faster than the other half, so the claim in the figure caption title is accurate. We edited the Fig. 3I caption for clarity. Examples can be seen in 3G (red) and 3H (blue).

(see also below – text describes results in that same figure in a completely different way...)

Statement on faster learning with presynaptic plasticity is not well substantiated. Even in 3F – it depends how learning is defined; if it's defined as a separation of synaptic weights in early vs late groups, then building up of the separation will be about same (at least looks like in this resolution). It also might depend on temporal difference between early and late inputs.

Again, it is not meaningful to directly compare the numerical results obtained with the latency and the correlation paradigms.

For the correlation paradigm, 3I clearly shows that it's a matter of input parameters.

Yes, we agree that Fig. 3I shows that it is matter of the parameters of the firing pattern. However, we want to clarify that model parameters are kept fixed across panels Fig. 3G-I.

Which scenario is more 'typical' depends on which combination(s) of input frequency and correlation are more typical for actual neuronal activity.

Yes, that is correct. For the parameter space we explored, the outcome was that typically, although not always, presynaptically expressed plasticity promoted faster learning, like we stated in the figure caption title. The figure caption text was edited for clarity.

l. 130-133. "This can be framed as a consequence of potentiation requiring glutamate release [50], so that in a more reliable synapse, with a high p value, there is a greater propensity for potentiation. Conversely, depression was slower with presynaptically expressed plasticity, again because lowered probability of release effectively also led to less plasticity"

Not clear what glutamate release has to do with plasticity in the model. As far as I understand, no mechanisms specific to glutamate release or glutamate receptors were implemented.

It is not about mechanism as much as it is about simple Hebbian pairing of pre- and postsynaptic activity during the induction. Glutamate release was used as synonymous to a successful spike transmission. A presynaptic spike that in an individual trial fails to cause release at a synapse due the probability of release does not satisfy the conditions for Hebbian learning in that trial, because in this trial scenario, the presynapse in fact fails to be active. We would hesitate to call this a mechanistic implementation in the model, because it is no different from solely spiking the postsynaptic cell during a trial and observing that no incremental plasticity was elicited in that trial.

l. 143-145. "...in a scenario in which there is competition due to e.g. limited resources, inputs with presynaptic plasticity would be expected to overcome inputs with postsynaptic plasticity"

What if presynaptic resources are limited? e.g. Small ready-to-release pool or slow replenishment of vesicles?

This is true, this argument does not really make good sense. We removed this confusing rationale.

l. 148 " Because of normalization, these two input populations competed, so that one potentiated at the expense of the oher, which depressed."

First, what normalization? Methods do not say a word about it.

The methods have been rectified. As this paradigm was quite unstable with the Song & Abbott model (see e.g., ref. 47, Babadi & Abbott, 2016), there were expected differences with the introduction of stochastic transmission (Song & Abbott model was deterministic) and varying higher statistics among the correlated inputs. Therefore, even though it achieved potentiation of correlated inputs in all cases, not all cases depressed the random inputs. For a symmetric comparison, we introduced a limit to the summed weights, which adjusted the 'learning' of correlated inputs to the competitive scheme seen in the original Song & Abbott. This did not affect which side 'won', since potentiation happened without this limit as well.

Second, how to reconcile "one population potentiated one depressed" with "which side potentiated faster" (Legend Fig 3I)? Slower potentiation is quite different from a depression.

Good point. The Fig. 3I caption and the main text were edited to clarify.

l. 154 and further – "Presynaptic expression modelled as changes in short-term plasticity".

Does this mean that 'presynaptic' model described so far did not include short-term plasticity? If yes, what is rationale for using such a model?

This is correct. See our above reply to point 2 for details. Briefly, the text was edited in several places to clarify this and to provide a rationale.

Fig. 4.

4E – what determined different steady-state of potentiated synapses in models with presynaptic (red) compared to postsynaptic or mixed (blue, black) expression?

Inputs that fire before the postsynaptic neuron get potentiated, and the ones that fire after get depressed. However, as the postsynaptic latency decreases, some inputs that fired just before the postsynaptic response change to just after and are consequently depressed. Since the number of inputs that correspond to the before or the after groups changes through the simulation, we separated them instead according to their position within the stimulus. 'Early' inputs fire in the first half of the stimulation, 'late' one in the second. At the start of the simulation this corresponds more or less to the before/after groups. As the latency shifts, some of the initially 'before' inputs get depressed within the 'early' group, which decreases the average value. We did not include this explanation in the manuscript, we are only stating it here, but we are happy to include it if the reviewer suggests so.

4A-E, latency paradigm – would the rate of change (and the difference between models) depend on specifics of input patterns?

The paradigm consists of inputs that are distributed normally in relation to a point in time. Parameters of the inputs are average intra-burst frequency and activity duration, as well as the width of delay distribution. Rates of plasticity would change — in the Song & Abbott model, plasticity is faster for higher input frequencies. However, in order to have P resulting in more change than q with STP, this would only happen if there were facilitating synapses. We did not include this explanation in the manuscript, we are only stating it here, but we are happy to include it if the reviewer suggests so.

l. 176. "... synaptic efficacy was still potentiated faster and depressed slower in the presynaptic case (Fig. 4E). " Not sure this is correct description. Red/early do potentiate more in the end, but rate of potentiation is about same as in black or blue, esp in the beginning.

Depression of 'late' looks pretty much the same in all three cases.

We added an inset with slope measures to Fig4E to clarify this point.

l. 185 In the simulations...

Fixed.

l. 198. "... in this model, LTP is expressed both pre- and postsynaptically, whereas LTD is solely postsynaptically expressed."

Fixed. It should have said "LTD is solely presynaptically expressed."

l. 211. " postsynaptic plasticity in the tuned model lacked the capacity to depress."

How the above two statements fit together?

There was a typo on line 198; line 211 is correct.

and next, l. 214 "the inclusion of presynaptic LTD"...

This is correct.

Please explain clearly which model included what; and if 'biologically tuned' model does not include presynaptic LTD, why is it still included here?

Lines 212-214 in the revised manuscript spell out which forms of plasticity were found in the biologically tuned model. Yes, the biologically tuned model does have presynaptic LTD; there was a typo on line 198 in the previous manuscript.

l. 218. "groups of correlated and uncorrelated inputs clustered (Fig. 6A) without the need for added competition through weight normalization" .

Does this mean that simulations were run with and without weight normalization? Please clarify in the methods, how normalization was implemented, and how it was turned on and off. In Results and Figures, please clearly indicate which results were obtained with model(s) with normalization, and which in models without normalization.

There was normalization included only on Figs 3I and 4G, the pre x post "competition" figures for Song & Abbott model. The text has been clarified.

l. 223-225 (and Fig 6C). Unclear, please explain.

A linear classifier was used to determine whether we could predict the input type given the average and variance values. The text and methods have been edited for clarity.

l. 226. "The presynaptic frequency range for optimal separation was between 50 and 80 Hz (Fig. 6C). At the other end of the range..."

What is 'the other end' of 50-80Hz range ???

Sorry, this was a typo, it was supposed to say, "at the lower end of the range". The manuscript was revised accordingly.

l. 234. to to

Fixed.

l. 235,236 "In combination with postsynaptic plasticity, presynaptic plasticity provided a degree of output control..." - not clear, please explain.

This sentence was rephrased in the revised manuscript.

Fig 5 Legend does not correspond to panel lettering.

Sorry, this was fixed.

l. 248 that that

Thanks, this was changed to "that the".

l. 262. "Presynaptic plasticity expressed as the regulation of release probability alone did not result in any differences over average postsynaptic activity measurements compared to postsynaptic expression."

Fig 3 or related text do not show any data on average postsynaptic activity in correlation paradigm.

We were referring to the latency paradigm, Figs. 3B and 4B, but the reference to Fig. 3B was somehow omitted. In the revised manuscript, the reference to Fig. 3B has been included and the text was clarified.

Methods.

Please define ALL parameters and terms in equations.

We have revised the Methods section to clarify this.

l.375-386. synaptic weight changes.

May be I am wrong (please correct/explain then), but unless you define qi= (0,1], qmax=Pmax =sqrt(qi) could be nonsense (say with qi=4).

We clarified this point in the revised manuscript. The weight q_i was indeed defined in the unit interval $q_i \in (0,1]$. Separately, q_{max} referred to the maximal amplitude that re-scaled the input to the conductance equation: q_i*q_{max}.

l. 384. "The largest possible change for P or q separately was tot = 1 − qi."

This implies that q close to 1 limits changes in P; Why P can't change if qi=1?

It was not that q limited P, just that P and q were always initialized to the same values. So, if q_i=1, then P_i=1. If the initial values are already maximized, then there can be no learning. This was clarified in the revised manuscript, lines 408-412.

l. 385-386 reads like bounds on P and q were set individually for different simulations; if so, please state this clearly and provide values of these limits for each simulation.

Sorry, this was not really the case. Rather, $P \in (0,1]$ and $q \in (0,1]$ in all simulations. What was set was the maximal amount of weight change, which depended on the initial value of the weights. All latency simulations were initialized at $p_i = q_i = 0.4$ and all correlation simulations at $p_i = q_i = 0.5$. The reason for $p_i = q_i = 0.4$ in the latency configuration was simply to achieve a wider latency reduction (as the initial postsynaptic latency would be later than if it was $p_i = q_i = 0.5$), and thus make it more visible in the graphs. This, however, does not change the interpretation of the simulations.

l. 405. "Presynaptic control of the probability of release per stimulus was implemented either as a Markovian process or as short-term plasticity."

STP can be implemented using a Markovian process too, also at 5 release sites with initially uniform P. I am not suggesting the authors to do so; but if this should mean that no STP was implemented, it should be clearly stated.

Thanks! We were indeed unclear in the previous version of the manuscript. This is now stated in several places in the revised manuscript, e.g., lines 86-115, and again on lines 118-120, etc.
