# Supplementary material for: Pre- and postsynaptically expressed spike-timing-dependent plasticity contribute differentially to neuronal learning
Source: PLoS Comput Biol. 2022 Jun 14;18(6):e1009409. doi: 10.1371/journal.pcbi.1009409 (PMC9236267; doi:10.1371/journal.pcbi.1009409)
Supplement: Attachment — Submitted filename: Mizusaki_Rebuttal.pdf [file pcbi.1009409.s003.pdf]

Rebuttal for Mizusaki *et al*, PCOMPBIOL-D-21-01572R1

We were elated to learn that Reviewers #1 and #2 were completely satisfied and that Reviewer #3 only had relatively minor concerns left for us to address. We have in our revised manuscript fully addressed all concerns. We are very grateful to all reviewers for their detailed and thoughtful feedback, which considerably strengthened our paper.

Reviewer #1

The paper is much clarified now.

We thank the reviewer for their positive feedback and for their detailed help.

Reviewer #2

The authors have addressed all of my concerns.

We are ever so grateful for the reviewer's helpful comments and for their positive feedback.

Reviewer #3

In the revised paper the authors addressed many of the concerns from previous comments.

We thank the reviewer for their thoughtful feedback and for taking their time with improving our paper.

However, I think a number of concerns remains not fully addressed.

Some parts of the text are still confusing (or have ambiguous meaning, implying what the authors probably did not mean to say), or plainly not correct.

Just few examples.

Introduction (which is by the way lengthy and lacks clear logics)

"In the 1990s, this led to a heated debate on the precise locus of expression of LTP, with some arguing for postsynaptic expression, whereas others were in favour of a presynaptic locus of LTP [8] (Fig. 1A). Beginning in the early 2000's, this controversy was gradually resolved by the realisation that plasticity depends critically on several factors, notably animal age, induction protocol, and precise brain region [9–11]. Indeed, this resolution has now been developed to the point that it is currently widely accepted that specific interneuron types have dramatically different forms of long-term plasticity [12, 13], meaning that long-term plasticity in fact depends on the particular synapse type [14]."

In the above, 1st sentence is about locus of expression;

2nd says controversy resolved, but talks about plasticity in general, without mentioning induction or expression or other features

3rd – all of a sudden talks about interneurons, while most of the debate on expression locus was about excitatory transmission to pyramidal neurons, mostly in the hippocampus...

We thank the reviewer for this feedback. We shortened the Introduction and elaborated specific parts, such as the above paragraph, as requested. We clarified that the pre-vs.-post debate focused on hippocampal CA1 LTP.

Also, why interneurons where some peculiar forms of plasticity were reported in 2007 and later, and not a really classical paper from 1990 on pre and postsynaptic forms of plasticity in CA3 neurons (Zalutsky, Nicoll 1990), or clear demonstration of presynaptic mechanisms in neocortical neurons (Markram, Tsodyks 1995)?

I mean, the intro makes an impression that authors care about history of the problem.

We thank the reviewer for this suggestion. To provide a more nuanced view on the pre-vs.-post debate, we now cite Zalutsky & Nicoll (1990) in the Introduction.

By Markram & Tsodyks (1995), we assume the Reviewer means Markram & Tsodyks (1996), which indeed is a classic, and we already cited it prominently. However, Markram & Tsodyks (1996) famously never claimed that the redistribution of synaptic efficacy they found was necessarily due to a presynaptic mechanism (see for example "*The most likely mechanism for increased…* on page 809 of that paper), thereby cleverly skirting around the pre-vs.-post debate. We can thus not cite Markram & Tsodyks (1996) to support a claim of presynaptic mechanisms in neocortical neurons. We do, however, cite it in support of the view that long-term plasticity can alter short-term plasticity (lines 51-51) and in support of redistribution of synaptic resources in a presynaptic modification scenario (lines 161-163).

"This is because presynaptically expressed plasticity leads to changes in synaptic dynamics, whereas postsynaptic expression does not (Fig.1B). For instance, during high-frequency bursting, as the readily releasable pool of vesicles in a synaptic bouton runs out, leading to short-term depression of synaptic efficacy [29], while short-term facilitation dominates at other synapse types [30]."

1st sentence – yes, OK.

2nd – this is not an elaboration of the statement in the previous sentence; also, readily releasable pool is also not infinite but is eventually depleted in synapses with PPF.

We thank the reviewer for approving of the first sentence. Regarding the second sentence, we added the word "may" as a qualifier, hopefully this addresses the point raised.

"As a corollary, it follows that presynaptic expression of plasticity may change the computational properties of a given synaptic connection."

Why postsynaptic increase (or decrease) is NOT a change of computational property of a synapse? Yes, it does not change the filtering properties as presynaptic changes would, but it will change the probability of that input inducing a postsynaptic spike (well, on background of other synaptic activity) – why is this NOT a computational property of a synapse?

We removed this sentence.

"In this case, increasing the probability of release by the induction of LTP"

This implies that p can change only one way – increase, which is not true.

The sentence was reworded.

"Experimentally, it is long known that the induction of neocortical long-term plasticity may alter short-term depression [16, 41]."

Can it also change short-term facilitation? Yes, many neocortical synapses show PPD, but some do show PPF (and in the hippocampus PPF is typical for many synapses).

I mean, the paper is not specifically about depressing, nor about specifically neocortical synapses, right?

We added "for example" to clarify.

(Discussion).

"Presynaptically expressed plasticity alone was not ideally suited for rate coding, because it did not impact the average summed input effectively. "

This would be kind of true ONLY for the case of exclusively-bursting activity; It is definitely NOT true for the case of low-frequency spikes; also, in case of irregular firing changing p would change steady-state amplitude.

Also, plasticity and coding are two different 'dimensions'; plasticity can change coding, or mediate encoding of learned information, but plasticity is NOT coding;

We changed the wording to "not ideally suited for changing rate coding", since we are dealing with plasticity.

"As a consequence, postsynaptic firing frequency remained relatively unchanged after presynaptically expressed plasticity (e.g., Figs. 4D, 5D, 6B)."

4D: about 15-20% increase of frequency, and about 40% decrease of burst duration; so kind of yes, total number of spikes in all bursts changed little. This does not say much about firing frequency, calculated using all spikes.

5D: about 50% decrease of frequency in bursts, and about 60-80% decrease in burst duration;

-> about 5 – 10 fold (!) decrease of spikes in bursts. Unless there is a compensatory increase of single spike (I did not find such data in the Results), this does not look to me as "relatively unchanged".

6B – yes.

Thus, in the three groups of results, one example supports the claim, one does not, and one may be...

Thank you for pointing this out. We forgot to remove two of the figure references in this part of the discussion. We apologize for the confusion, and have now rephrased the text as follows:

Presynaptically expressed plasticity alone was not ideally suited for changing rate coding, because presynaptic short-term plasticity acts as a filter on the presynaptic firing rate. As a consequence, postsynaptic instantaneous firing frequency shows reduced changes (Figs. 4D, 5D) or no changes (Fig. 6B) when compared to postsynaptic plasticity.

These are just some examples, there are many more in the text.

Here, it is unclear what specific changes the reviewer is asking for.

Bottom line: I would strongly recommend the senior authors to carefully read/edit the text, paying attention to preserving logical connections between sentences, overall consistency when referring to results of prior experimental and theoretical work, and consistency of interpretation of and conclusions from their own results.

We thank Reviewer #3 for their advice. We think that now the logical connections between the different parts are clearer in the revised manuscript.
